# Epidemiological characteristics of anthropophilic and zoophilic dermatophytosis: a 40-year retrospective prevalence study

**DOI:** 10.1186/s40249-026-01469-y

**Published:** 2026-07-01

**Authors:** Freddy Villanueva-Cotrina, Vilma Bejar, Víctor Raul Huaman-Cardenas, Katherine Yauri Condor, Jose Guevara

**Affiliations:** 1 Mycology Laboratory, Institute of Tropical Medicine “Daniel Alcides Carrion” - National University of San Marcos, Lima, Peru; 2 Mycology Laboratory, Institute of Regional Medicine - National University of the Northeast. CONICET, Resistencia, Chaco Argentina; 3 School of Medical Technology, Continental University, Lima, Peru

**Keywords:** Cutaneous mycoses, Tinea, Fungal ecology niche, Peru

## Abstract

Dermatophytosis is commonly caused by anthropophilic or zoophilic fungi. Although factors related to the disease onset have been identified, it is unclear whether these factors influence disease prevalence differently depending on the fungus’s ecological niche. Our study aimed to determine the epidemiological characteristics of dermatophytosis caused by anthropophilic versus zoophilic agents. We conducted a retrospective descriptive study (1976–2015) including confirmed cases of dermatophytosis diagnosed by positive mycological cultures in patients treated at the Institute of Tropical Medicine “Daniel Alcides Carrion”—National University of San Marcos, Lima, Peru. Demographic and microbiological data were collected. We used the Chi-square test to evaluate differences in the prevalence of anthropophilic and zoophilic dermatophytosis across the variables of interest. A total of 4961 cases of dermatophytosis were diagnosed. Anthropophilic dermatophytosis was most prevalent in adults, primarily due to *Trichophyton rubrum* (92.19%) and *T. interdigitale* (94.16%) (*P* < 0.001). In contrast, zoophilic dermatophytosis was most prevalent in children, primarily due to *Microsporum canis* (64.24%) (*P* < 0.001). Furthermore, anthropophilic dermatophytosis was most prevalent in tinea unguium due to *T. rubrum* (61.49%) and *T. interdigitale* (62.12%), as well as in tinea pedis due to *T. interdigitale* (37.88%) (*P* < 0.001). Conversely, zoophilic dermatophytosis was most prevalent in tinea capitis due to *M. canis* (54.88%), as well as in tinea manum and tinea corporis due to *T. mentagrophytes* (25.71% and 37.71%, respectively) (*P* < 0.001). According to its ecology, dermatophytosis was related to the age group affected, the clinical form presentation, and a specific etiology.

**Graphical Abstract:**

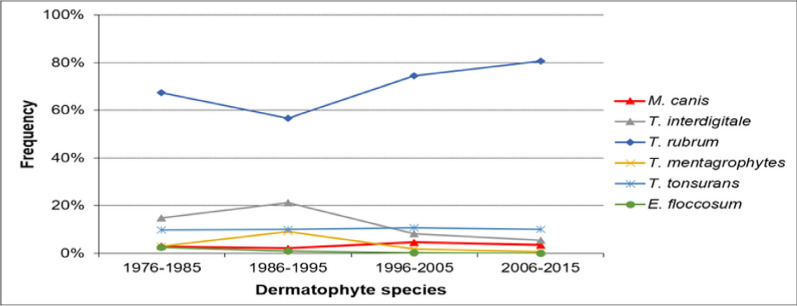

## Background

Dermatophytosis is the most prevalent fungal infection worldwide [1–4]. The fungal species responsible for this infection belong to the genera *Trichophyton*, *Microsporum*, *Epidermophyton, Nannizzia, Paraphyton, Lophophyton, Arthroderma, Ctenomyces*, and *Guarromyces*. The genera *Trichophyton*, *Microsporum*, and *Epidermophyton* are the most commonly isolated and colonize human and animal hosts [5–9]. *T. rubrum*, *T. interdigitale*, *T. tonsurans*, *E. floccosum*, and *T. violaceum* are classified as anthropophilic fungi that primarily infect humans and only rarely affect animals. In contrast, *T. mentagrophytes*, *M. canis*, and *T. verrucosum* are categorized as zoophilic fungi and are essentially animal pathogens but can also infect humans [2, 10].

Dermatophytes invade and proliferate in keratinized tissues, such as the skin, scalp, hair, and nails, resulting in scaling and annular lesions with inflammatory, erythematous, and pruritic borders [10]. These cutaneous mycoses present in various clinical forms known as tinea, which are classified according to the affected anatomical site. The most common presentations are tinea unguium (nail infection) and tinea pedis (foot infection) [2, 10]. Tineas typically exhibit a characteristic etiology such as *T. rubrum* predominantly in tinea unguium and tinea pedis [5, 6, 11], and *T. tonsurans* in tinea capitis [11, 12].

Several recognized factors influence the onset and persistence of dermatophytosis. Tropical and subtropical climates, which are characterized by high temperatures and humidity, facilitate the proliferation of the causative agents [13, 14]. Correspondingly, while these mycoses affect various anatomical regions, the fingernails and toenails are the most frequently affected sites [15, 16]. Furthermore, certain demographic characteristics are associated with the development of dermatophytosis. While these infections affect all age group, with a higher prevalence typically observed in adults [7, 17], significant prevalence rates have also been reported in pediatric populations [18]. Similarly, while some studies identify males as the most frequently affected group [5, 19], others have reported a higher prevalence among females [17, 20].

Dermatophytosis presents diverse clinical forms with different etiologies, and its prevalence varies according to associated developmental factors. Therefore, it is essential to determine if there is a pattern of species distribution among tineas and if factors influence disease prevalence differently based on the fungus’s ecological niche. This study aimed to determine the epidemiological characteristics of dermatophytosis caused by anthropophilic versus zoophilic agents. Additionally, the study sought to determine their prevalence and longitudinal trends over the four decades it covered.

## Methods

### Study design, area and population

This is a descriptive, retrospective study. It was conducted in the Bellavista district, which is located in the central coastal region of Callao Province in Lima, Peru. Bellavista is a densely populated urban area categorized as middle and lower-middle class, with a subtropical desert climate and extreme relative humidity levels [21, 22]. The study population comprised individuals residing in and around urban districts who presented with clinical and epidemiological signs of dermatophytosis, and who were admitted to the dermatology department at the Institute of Tropical Medicine “Daniel Alcides Carrion”—National University of San Marcos, Lima, Peru between 1976 and 2015. A diagnosis of dermatophytosis was established by isolating the dermatophyte pathogen in a positive mycological culture.

### Data collection and study variables

Demographic and microbiological data were collected from clinical records and laboratory logs at the institute. The demographic variables analyzed included season (summer, autumn, winter, and spring) [23], age group (children and adults), and gender. Laboratory variables included the anatomical site, the type of specimen processed, and the specific etiology. The laboratory study involved the direct examination and culturing of skin scales, nails, and hair samples obtained from various anatomical sites that were suspected of having tinea lesions. Fungal identification was performed using macroscopic and microscopic morphological characterization [24]. Dermatophytosis cases were recorded and categorized by decade for longitudinal analysis. Additionally, data on mean temperature and precipitation (in rainy days) for each season across decades were recorded [25].

### Nomenclature of the *Trichophyton mentagrophytes* complex

Previous studies utilizing sequencing of the 28S rRNA gene and internal transcribed spacer (ITS) regions [26–28], as well as mating experiments [29] have enabled a clear distinction between the two species within the *Trichophyton mentagrophytes* complex, and their correlation with the affected anatomical sites. Consequently, isolates recovered from tinea pedis and tinea unguium were classified as *T. interdigitale*, while isolates from other sites were categorized as *T. mentagrophytes*.

### Statistical analysis

The data frequencies of the categorical variables were determined using absolute values and percentages. The Chi-square test was used to evaluate differences in the prevalence of anthropophilic and zoophilic dermatophytosis across the categorical variables of interest. Changes in the prevalence of these dermatophytosis over the study periods were assessed, with a significance level of *P* < 0.01 established for all tests. Data analysis was performed using Stata version 14.0 software (StataCorp, College Station, Texas 77845 USA). To ensure taxonomic coherence and statistical robustness in evaluating long-term endemic trends, cases of dermatophytosis involving a species with a relative frequency below 0.1% were excluded from the final dataset.

## Results

A total of 17,165 patients with suspected dermatophytosis were evaluated throughout the study period. Of these, 4964 positive cultures were identified; however, three cases (0.06%) involving rare species such as *T. verrucosum* (two cases) and *T. violaceum* (one case) were excluded. Consequently, the diagnosis was confirmed and analyzed in 4961 cases (28.90%). Of these, 4619 (93.11%) were caused by anthropophilic dermatophytes and 342 (6.89%) by zoophilic species. Regarding the climate, data shows that temperatures were remained consistent, with no more than a 1 °C difference between decades for each season. Additionally, the data indicates that precipitation was scarce throughout the four-decade study period. (Table 1).

**Table 1:** Seasonal averages of temperature and precipitation in Lima, Peru between 1976 and 2015

Season	1976–1985	1986–1995	1996–2005	2006–2015
Temperature (°C)				
Summer	24.14	23.55	24.05	24.57
Autumn	20.23	19.58	19.97	19.84
Winter	16.55	15.69	16.51	16.04
Springer	19.65	19.08	19.76	19.10
Precipitation (mm)				
Summer	0.0005	0.0579	0.0492	0.0120
Autumn	0.0178	0.0296	0.0072	0.0268
Winter	0.0296	0.1042	0.0350	0.1399
Springer	0.0010	0.0286	0.0018	0.0091

### Baseline characteristics of dermatophytosis

Over the 40-year study period, diagnoses of dermatophytosis increased steadily, rising from 15.50% in the first decade (1976–1985) to 27.55% in the final decade (2006–2015). The highest prevalence of cases was observed during the summer (35.46%) and in the adult age group (≥ 16 years), accounting for 83.84% of the population. A slightly highest frequency was observed in males (51.05%). The feet were markedly the most affected anatomical region (62.93%), followed by the hands (10.79%) and the scalp (8.28%). Furthermore, over half of the cases were diagnosed from nail specimens (51.55%), and the most frequently isolated etiological agent was *T. rubrum* (70.37%). The most prevalent clinical forms were tinea unguium (51.70%) and tinea pedis (18.38%). Anthropophilic dermatophytes were the most prevalent (93.11%), and direct examination findings correlated with culture results in 71.43% of cases. (Table 2).

**Table 2:** Epidemiological characteristics of 4961 cases of dermatophytosis at the Institute of Tropical Medicine “Daniel Alcides Carrion”—National University of San Marcos, Lima, Peru between 1976 and 2015

Characteristics	*N* (%)
Decade	
1976–1985	770 (15.50)
1986–1995	1282 (25.84)
1996–2005	1542 (31.08)
2006–2015	1367 (27.55)
Season of the year	
Summer	1759 (35.46)
Autumn	1164 (23.46)
Winter	1007 (20.30)
Spring	1031 (20.78)
Age groups (years)	
0–15	793 (16.16)
≥ 16	4115 (83.84)
Gender	
Male	2531 (51.05)
Female	2427 (48.95)
Anatomical site	
Scalp	409 (8.28)
Face	142 (2.88)
Neck	38 (0.77)
Armpit	7 (0.14)
Chest	40 (0.81)
Arm	81 (1.64)
Hand	533 (10.79)
Back	39 (0.79)
Abdomen	32 (0.65)
Groin	272 (5.51)
Buttock	85 (1.72)
Thigh	64 (1.30)
Leg	89(1.80)
Foot	3108 (62.93)
Sample	
Nails	2557 (51.55)
Skin scales	1988 (40.08)
Scalp and/or hair	415 (8.37)
Etiology	
*Trichophyton rubrum*	3491 (70.37)
*T. interdigitale*	586 (11.81)
*T. tonsurans*	510 (10.28)
*Epidermophyton floccosum*	32 (0.65)
*T. mentagrophytes*	177 (3.57)
*Microsporum canis*	165 (3.33)
Tinea	
*T. capitis*	409 (8.27)
* T. faciei*	142 (2.87)
*T. cruris*	272 (5.50)
*T. manum*	188 (3.80)
*T. corporis*	469 (9.48)
*T. pedis*	909 (18.38)
* T. unguium*	2557 (51.70)
Ecological niche	
Anthropophilic	4619 (93.11)
Zoophilic	342 (6.89)
Direct examination	
Positive	3544 (71.44)
Negative	1417 (28.56)

^*^Three additional cases (< 0.1% of total) were identified but excluded from the trend analysis: two isolates of *T. verrucosum* and one isolate of *T. violaceum*

### Trends in dermatophytosis prevalence according to ecological niche

**Fig. 1:**
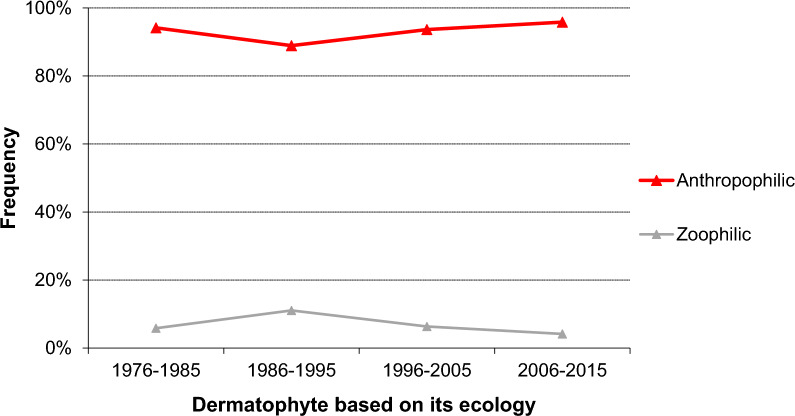
Dermatophytosis prevalence according to ecological niche at the Institute of Tropical Medicine “Daniel Alcides Carrion”—National University of San Marcos, Lima, Peru between 1976 and 2015

The prevalence of anthropophilic dermatophytosis was markedly higher than zoophilic dermatophytosis, exceeding 93.00% across all four decades. However, an exception occurred during the period from 1986 to 1995, when the prevalence declined to 88.92%. This coincided with a significant increase in zoophilic dermatophytosis, which reached their highest prevalence of 11.08% (*P* < 0.001). (Fig. 1).

**Fig. 2:**
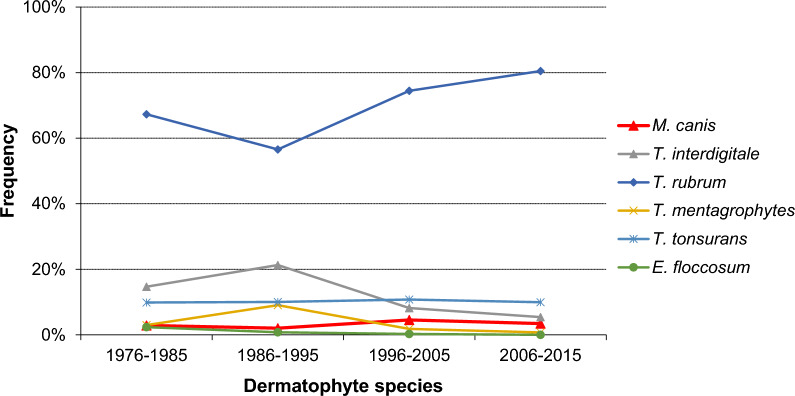
Distribution of dermatophyte species prevalence at the Institute of Tropical Medicine "Daniel Alcides Carrion"—National University of San Marcos, Lima, Peru between 1976 and 2015

The temporal distribution of the species involved in these prevalence shifts shows that *T. rubrum* was the dominant anthropophilic species throughout the study. Its prevalence started at 67.27%, decreased to 56.55% in the 1986–1995 period, and increased to 80.47% by the end of the study period. Conversely, *T. interdigitale* increased during the same period, reaching 21.29%, before decreasing to a final prevalence of 5.41% in subsequent decades. Among the zoophilic species, *T. mentagrophytes* was the most prevalent, peaking at 9.05% during the 1986–1995 period. However, it subsequently declined abruptly to 0.73% and was surpassed by *M. canis* in the last two decades. Finally, *T. tonsurans* and *E. floccosum* consistently maintained prevalence rates consistently below 10% across all four decades studied (*P* < 0.001). (Fig. 2).

### Epidemiological characteristics of dermatophytosis according to ecological niche

Anthropophilic dermatophytosis prevailed over zoophilic infections across all four seasons, accounting for over 91.00% of cases. No significant seasonal differences were detected (*P* = 0.133). Anthropophilic dermatophytosis was most prevalent in adults (94.87% versus 83.48%), whereas zoophilic dermatophytosis was most prevalent in children (16.52% versus 5.13%) (*P* < 0.001). No significant differences were observed in the distribution of dermatophytosis ecology according to gender (*P* = 0.084). Regarding specimen types, anthropophilic dermatophytosis were predominantly diagnosed in nail samples (99.96%), while zoophilic dermatophytosis were more prevalent in scalp samples (25.30%) (*P* < 0.001). Similarly, anthropophilic dermatophytosis primarily affected the feet (99.84%) and hands (90.81%), whereas zoophilic infections were more prevalent on the thorax (32.50%) and arms (32.10%) (*P* < 0.001). Anthropophilic dermatophytosis primarily manifested as tinea unguium (99.96%) and tinea pedis (99.56%), while zoophilic dermatophytosis were more frequently associated with tinea capitis (25.18%), tinea faciei (26.06%), tinea manum (26.06%), and tinea corporis (23.45%) (*P* < 0.001). (Table 3).

**Table 3:** Epidemiological characteristics of 4961 cases of dermatophytosis according to ecological niche at the Institute of Tropical Medicine “Daniel Alcides Carrion”—National University of San Marcos, Lima, Peru between 1976 and 2015

Variables	Anthropophilic	Zoophilic	*P* value
	*N* (%)	*N* (%)	
Season of the year			
Summer	1653 (93.97)	106 (6.03)	0.133
Autumn	1086 (93.30)	78 (6.7)	
Winter	935 (92.85)	72 (7.15)	
Spring	945 (91.66)	86 (8.34)	
Age groups (years)			
0–15	662 (83.48)	131 (16.52)	< 0.001
≥ 16	3904 (94.87)	211 (5.13)	
Gender			
Male	2341 (92.49)	190 (7.51)	0.084
Female	2275 (93.74)	152 (6.26)	
Sample			
Nails	2556 (99.96)	1 (0.04)	< 0.001
Skin scales	1752 (88.13)	236 (11.87)	
Scalp and/or hair	310 (74.70)	105 (25.30)	
Anatomical site			
Scalp	306 (74.82)	103 (25.18)	< 0.001
Face	105 (73.94)	37 (26.06)	
Neck	32 (84.21)	6 (15.79)	
Armpit	5 (71.43)	2 (28.57)	
Chest	27 (67.50)	13 (32.50)	
Arm	55 (67.90)	26 (32.10)	
Hand	484 (90.81)	49 (9.19)	
Back	29 (74.36)	10 (25.64)	
Abdomen	27 (84.13)	5 (15.63)	
Groin	237 (87.13)	35 (12.87)	
Buttock	75 (88.24)	10 (11.76)	
Thigh	49 (76.56)	15 (23.44)	
Leg	64 (71.91)	25 (28.09)	
Foot	3103 (99.84)	5 (0.16)	
Tinea			
*Trichophyton* *capitis*	306 (74.82)	103 (25.18)	< 0.001
*T. faciei*	105 (73.94)	37 (26.06)	
* T. cruris*	237 (87.13)	35 (12.87)	
* T. manum*	139 (73.94)	49 (26.06)	
* T. corporis*	359 (76.55)	110 (23.45)	
*T. pedis*	905 (99.56)	4 (0.44)	
* T. unguium*	2556 (99.96)	1 (0.04)	

Furthermore, the distribution of species was analyzed according to ecological niche for the variables in which significant differences in dermatophytosis prevalence were previously observed, such as age group and clinical form. The highest prevalence of anthropophilic dermatophytosis in children was primarily caused by *T. tonsurans* (69.74%), while in adults it was primarily due to *T. interdigitale* (94.16%) and *T. rubrum* (92.19%) (*P* < 0.001). Conversely, the highest prevalence of zoophilic dermatophytosis in children was primarily caused by *M. canis* (64.24%), whereas in adults it was primarily due to *T. mentagrophytes* (85.88%) (*P* < 0.001). (Table 4).

**Table 4:** Distribution of anthropophilic and zoophilic dermatophyte species according to age group at the Institute of Tropical Medicine “Daniel Alcides Carrion”—National University of San Marcos, Lima, Peru between 1976 and 2015

Dermatophyte	0–15 years, *n* (%)	≥ 16 years, *n* (%)	*P* value
Anthropophilic			
*Trichophyton rubrum*	269 (7.81)	3174 (92.19)	< 0.001
*T. interdigitale*	34 (5.84)	548 (94.16)	
*T. tonsurans*	355 (69.74)	154 (30.26)	
*Epidermophyton floccosum*	4 (12.50)	28 (87.50)	
Zoophilic			
*T. mentagrophytes*	25 (14.12)	152 (85.88)	< 0.001
*Microsporum canis*	106 (64.24)	59 (35.76)	

On the other hand, the highest prevalence of the most common anthropophilic dermatophytosis, such as tinea unguium, were primarily caused by *T. interdigitale* (62.12%) and *T. rubrum* (61.49%). Meanwhile, tinea pedis was primarily attributed to *T. interdigitale* (37.88%) (*P* < 0.001). In contrast, the highest prevalence of the most common zoophilic dermatophytosis, such as tinea capitis, was primarily caused by *M. canis* (54.88%). Meanwhile, in tinea manum and tinea corporis, it was primarily caused by *T. mentagrophytes* (25.71% and 37.71%, respectively) (*P* < 0.001). (Table 5).

**Table 5:** Distribution of anthropophilic and zoophilic dermatophyte species according to clinical form (tinea) at the Institute of Tropical Medicine “Daniel Alcides Carrion”—National University of San Marcos, Lima, Peru between 1976 and 2015

Dermatophyte	*T. capitis,* *n* (%)	*T. faciei,* *n* (%)	*T. cruris,* *n* (%)	*T. manum,* *n* (%)	*T. corporis,* *n* (%)	*T. pedis,* *n* (%)	*T. unguium,* *n* (%)	*P* value
Anthropophilic								
*Trichophyton rubrum*	8 (0.23)	31 (0.89)	228 (6.55)	133 (3.82)	279 (8.02)	661 (18.99)	2140 (61.49)	< 0.001
*T. interdigitale*	0 (0)	0 (0)	0 (0)	0 (0)	0 (0)	222 (37.88)	364 (62.12)	
*T. tonsurans*	298 (58.43)	74 (14.51)	1 (0.20)	6 (1.18)	73 (14.31)	13 (2.55)	45 (8.82)	
*Epidermophyton floccosum*	0 (0)	0 (0)	8 (25.81)	0 (0)	7 (22.58)	9 (29.03)	7 (22.58)	
Zoophilic								
*T. mentagrophytes*	13 (7.43)	16 (9.14)	35 (20)	45 (25.71)	66 (37.71)	0 (0)	0 (0)	< 0.001
*Microsporum canis*	90 (54.88)	21 (12.80)	0 (0)	4 (2.44)	44 (26.83)	4 (2.44)	1 (0.61)	

Finally, a significantly higher number of positive direct examinations were observed in the diagnosis of anthropophilic dermatophytosis (3322; 71.91%) than in the diagnosis of zoophilic dermatophytosis (222; 64.91%) (*P* = 0.006). (Table 6).

**Table 6:** Direct examination results in the diagnosis of dermatophytosis categorized by ecological niche at the Institute of Tropical Medicine “Daniel Alcides Carrion”—National University of San Marcos, Lima, Peru between 1976 and 2015

Variables	Direct examination		*P* value
	Negative	Positive	
Dermatophyte	*N* (%)	*N* (%)	0.006
Anthropophilic	1297 (28.08)	3322 (71.91)	
Zoophilic	120 (35.09)	222 (64, 91)	

## Discussion

Dermatophytosis is considered to be the most common zoonotic disease [30]. Close interaction between animals and humans is becoming more common over time, particularly in urban societies [31]. This increases the likelihood of dermatophytosis developing in individuals exposed to animals that are colonized or infected by dermatophytes [32, 33]. Therefore, studying dermatophytosis requires consideration of the pathogens’ habitats and the epidemiological characteristics of populations affected.

We found significant differences in the prevalence of anthropophilic and zoophilic dermatophytosis according to factors such as age group, clinical presentation, and causative agents. Our results suggest that these factors may influence the prevalence of dermatophytosis based on their ecology within our study population, which exhibits characteristics of socioeconomic vulnerability. Dermatophytosis is strongly associated with poor hygiene, overcrowding and limited access to healthcare. These factors create favorable conditions for the spread of fungi, making dermatophytosis a significant health issue in developing nations such as ours [34]. Conversely, climate factors such as temperature and precipitation, which have remained consistent over decades, seem to play a minor role in the prevalence of this fungal infection in our study population.

Over the course of the study, the prevalence of dermatophytosis shows a clear predominance of anthropophilic fungi over zoophilic species. This finding is consistent with the ecological and biological affinity of these pathogens for humans [35–37]. These factors have been extensively studied in various anthropophilic dermatophytes, particularly in *T. rubrum*, the most commonly isolated species globally [2]. This species is often associated with adverse socioeconomic conditions in developing countries [38], characteristics that were also present in our study population. However, the transient decline of *T. rubrum* during the second decade may have resulted from the introduction of new antifungal drugs in the 1980s, such as ketoconazole. This antifungal has proven effective against anthropophilic dermatophytes such as *T. rubrum*, yet its efficacy against zoophilic species is variable [39]. The subsequent resurgence of *T. rubrum* dominance after the 1980s may be associated with a reduction in ketoconazole use following reports of its hepatotoxicity [40]. Additionally, the emergence of resistant isolates cannot be ruled. Conversely, the increase in *T. mentagrophytes*, in the same decade, may be driven by factors such as an increase in pet ownership, which is a leading cause of dermatophytosis in pets in various countries [41]. Furthermore, the prevalent occurrence of zoophilic dermatophytosis in rural populations [42] and their subsequent migration to urban centers, as witnessed in Peru during the 1980s [43], could be linked to this trend.

Analysis of the distribution of dermatophyte species by age group reveals distinct etiologies, indicating a higher prevalence of anthropophilic dermatophytosis in adults and zoophilic dermatophytosis in children. Anthropophilic infections in adults, primarily caused by *T. rubrum* and *T. interdigitale*, are attributed to frequent human-to-human contact during routine activities in workplaces and high-traffic public venues. Additionally. the development of these dermatophytosis is also related to chronic diseases that predominantly affect adults. Diabetes impairs the skin's barrier function and reduces the local immune response. Hormonal factors, such as low testosterone levels, have also been linked to the emergence of anthropophilic dermatophyte infections [44–46]. Conversely, the high prevalence of zoophilic dermatophytosis in children may be due to increased exposure to these pathogens resulting from close and frequent contact during play or handling of pets [10, 47]. The high prevalence of *M. canis* as a zoophilic dermatophyte in our population is linked to its wide distribution of this fungus among common pets, such as cats and dogs [48]. In addition to direct contact with pets, exposure to their habitats such as soil, gardens and farms, may lead to zoophilic dermatophytosis development. These environments harbor fungal biota derived from animal dander or hair and serve as reservoirs for these pathogens [49].

The ecology of dermatophytosis is closely linked to the development of specific clinical forms. Anthropophilic dermatophytes predominated in tinea unguium and tinea pedis, whereas zoophilic dermatophytes were more prevalent in tinea capitis, tinea faciei, tinea manum, and tinea corporis. The high prevalence of anthropophilic dermatophytosis presenting as tinea unguium, associated with *T. rubrum* and *T. interdigitale* in our study, may be attributed to the anatomical and physiological characteristics of this region. The subungual space provides a moist and warm microenvironment, particularly in individuals whose habits favor perspiration creating ideal conditions for fungal sporulation and proliferation [13, 50]. Similarly, the high prevalence of tinea pedis can be explained by the fact that anthropophilic dermatophytes find favorable habitats in feet due to abundant keratin, persistent moisture, and temperatures that replicate optimal conditions for their survival and reproduction. Factors that increase heat and moisture in the feet, such as wearing closed footwear, excessive sweating, and poor hygiene, generate a microenvironment that promotes dermatophyte proliferation [51]. Conversely, the highest prevalence of zoophilic dermatophytosis due to *M. canis* in tinea capitis and tinea faciei, as observed in our study may be attributed to the biological and anatomical characteristics. Children are more susceptible to tinea capitis due to the absence or insufficient levels of sebaceous fatty acids, which naturally protect against fungal colonization. [52–54]. Furthermore, the face is a common infection site in children due to the anatomical proximity of the scalp (a frequent focus of tinea capitis) to the face, facilitating autoinoculation [55, 56]. The face may also be a common infection site in children due to actions such as petting, hugging, or sleeping with infected pets [55]. Additionally, we found that *T. mentagrophytes* was the predominant agent in cases of tinea manum and tinea corporis, which is consistent with previous studies [57, 58]. The highest prevalence of this zoophilic species in adults suggests that frequent hand exposure during work, whether through contact with human skin or animals, facilitates the acquisition of this infection.

We emphasize the need for a One Health approach when studying dermatophytosis in order to improve our understanding of how it emerges and spreads. Knowing the specific type of dermatophytosis based on its ecology is highly relevant because specific etiologies and clinical forms are associated with anthropophilic and zoophilic habitats, which has implications for therapeutic management [59]. Furthermore, continuous prevalence surveillance is necessary because the infection sources, transmission mechanisms, and affected populations can change over time [60]. This knowledge is essential for developing elimination strategies and effective treatment protocols [10].

Our study had some limitations that motivate further prospective research to address them. For instance, we did not evaluate factors associated with the development of dermatophytosis based on their ecology, such as occupation, pet ownership, and attendance at public places. However, our results, based on a large sample size over four decades, offer insights into the potential factors influencing the prevalence of anthropophilic and zoophilic dermatophytosis. Additionally, although we did not perform genetic studies, which are the gold standard for fungal identification, identifying fungi based on the morphological characteristics of isolates enables us to ascertain whether an isolate belongs to a recognized dermatophytes species, such as *T. rubrum*. This method is also affordable for laboratories with limited resources, such as ours.

In conclusion, the marked predominance of anthropophilic dermatophytosis prevalence over zoophilic dermatophytosis is consistent with the ecological and biological affinity of their causative agents for humans. Furthermore, the epidemiological associations between dermatophytosis, classified by ecology, and the age group affected, clinical presentation, and etiology, can be linked to human habits and their interaction with animals. Our study provides valuable epidemiological data for preventing and controlling dermatophytosis by emphasizing the habitats and potential infection sources of these pathogens.

## Data Availability

All data is included in the article.
